# High Cyclin E Staining Index in Blastemal, Stromal or Epithelial Cells Is Correlated with Tumor Aggressiveness in Patients with Nephroblastoma

**DOI:** 10.1371/journal.pone.0002216

**Published:** 2008-05-21

**Authors:** Dominique Berrebi, Julie Leclerc, Gudrun Schleiermacher, Isabelle Zaccaria, Liliane Boccon-Gibod, Monique Fabre, Francis Jaubert, Alaa El Ghoneimi, Cécile Jeanpierre, Michel Peuchmaur

**Affiliations:** 1 Assistance Publique-Hôpitaux de Paris (AP-HP), Hôpital Robert Debré, Service d'Anatomie et de Cytologie Pathologiques, Paris, France; 2 Université Paris 7, UFR médecine EA3102, Paris, France; 3 Inserm U574, Hôpital Necker-Enfants Malades, Université Paris Descartes, Faculté de médecine, Paris, France; 4 Institut Curie, Service d'Oncologie Pédiatrique, Paris, France; 5 Assistance Publique-Hôpitaux de Paris (AP-HP), Hôpital Robert Debré, Unité d'Epidémiologie Clinique, Paris, France; 6 Inserm, Hôpital Robert Debré, CIE5, Paris, France; 7 Assistance Publique-Hôpitaux de Paris (AP-HP), Hôpital Armand Trousseau, Service d'Anatomie et de Cytologie Pathologiques, Paris, France; 8 Assistance Publique-Hôpitaux de Paris (AP-HP), Hôpital Bicêtre, Service d'Anatomie et de Cytologie Pathologiques, le Kremlin-Bicêtre, Paris, France; 9 Assistance Publique-Hôpitaux de Paris (AP-HP), Hôpital Necker-Enfants Malades, Service d'Anatomie et de Cytologie Pathologiques, Paris, France; 10 Assistance Publique-Hôpitaux de Paris (AP-HP), Hôpital Robert Debré, Service de Chirurgie Infantile, Paris, France; Uppsala University, Sweden

## Abstract

**Purpose:**

Identifying among nephroblastoma those with a high propensity for distant metastases using cell cycle markers: cyclin E as a regulator of progression through the cell cycle and Ki-67 as a tumor proliferation marker, since both are often deregulated in many human malignancies.

**Methodology/Principal Findings:**

A staining index (*SI*) was obtained by immunohistochemistry using anti-cyclin E and anti-Ki-67 antibodies in paraffin sections of 54 postchemotherapy nephroblastoma including 42 nephroblastoma without metastasis and 12 with metastases. Median cyclin E and Ki-67 *SI* were 46% and 33% in blastemal cells, 30% and 10% in stromal cells, 37% and 29.5% in epithelial cells. The highest values were found for anaplastic nephroblastoma. A correlation between cyclin E and Ki-67 *SI* was found for the blastemal component and for the epithelial component. Univariate analysis showed prognostic significance for metastases with cyclin E *SI* in stromal cells, epithelial cells and blastemal cells (p = 0.03, p = 0.01 and p = 0.002, respectively) as well as with Ki-67 *SI* in blastema (p<10^−4^). The most striking data were that both cyclin E *SI* and blastemal Ki-67 *SI* discriminated between patients with metastases and patients without metastasis among intermediate-risk nephroblastoma.

**Conclusions:**

Our findings show that a high cyclin E *SI* in all components of nephroblastoma is correlated with tumor aggressiveness and metastases, and that assessment of its expression may have prognostic value in the categorization of nephroblastoma.

## Introduction

Nephroblastoma is the most common pediatric tumor of the kidney [1]. It arises from metanephric blastemal cells and recapitulates renal embryogenesis. In Europe, patients are treated according to the International Society of Pediatric Oncology (SIOP) protocol, which consists of preoperative chemotherapy and surgical resection followed by postoperative treatment [2]. This latter step is adjusted on the basis of tumor histology and local tumor stage. Stage I low-risk nephroblastoma receive no postoperative treatment while high-risk tumors (i.e. diffuse anaplasia and blastemal types) are treated with aggressive chemotherapy. In the intermediate-risk tumor group (i.e. epithelial, stromal, mixed, regressive and focal anaplasia types), over 90% of the patients are cured with the SIOP therapeutic strategy, but a small fraction of children will relapse or metastasize. Thus, there is still a need for accurate molecular prognostic markers to identify these intermediate-risk tumors that need more intensive treatment.

A vast amount of prognostic markers in nephroblastoma have been reviewed [3], [4] and no biological marker was found that provided consistent predictive information regarding the clinical outcome. Tumor-specific loss of heterozygosity (LOH) for chromosomes 1p or 16q has been shown recently to be associated with a poorer prognosis in favorable-histology Wilms tumor entered in NWTS-5 (National Wilms' Tumor Study 5) [5] and is the only biological marker with immediate implications for treatment in the current Children's Oncology Group (COG) study. However, the prognostic value of these LOH for patients treated with preoperative chemotherapy according to the European SIOP protocol remains to be evaluated.

Some markers, such as Ki-67, may be relevant for assessing proliferative activity [3]. Ki-67, a nuclear antigen associated with cell proliferation, is present throughout the cell cycle and absent in resting cells [6]. High Ki-67 is associated with a more aggressive clinical behavior, and is found to be a significant determinant of distant metastasis and tumor-related death in adult tumors [7].

Cyclin E is the regulatory subunit of the cyclin E–Cdk2 complex, which takes part in the control of progression through G1 phase. Its activity is tightly regulated during normal cell cycle. In neoplastic cells, deregulation is often observed and is thought to play a fundamental role in tumorigenesis [8]. Cyclin E overexpression has been studied and identified as an adverse prognostic marker in a wide variety of human adult cancers [9], [10], [11], [12]. However, to our knowledge, cyclin E levels have never been investigated in solid embryonal tumors, characterized by a high proliferation rate.

The aim of our study was to evaluate cyclin E expression in nephroblastoma using immunohistochemistry. To check if cyclin E overexpression reflects only increased proliferation, levels of the commonly used proliferation marker, Ki-67, were simultaneously assessed. Analysis of the results was carried out taking into account the global SIOP histology.

## Results

### Ki-67 and cyclin E expression in postchemotherapy nephroblastoma

**Figure 1:**
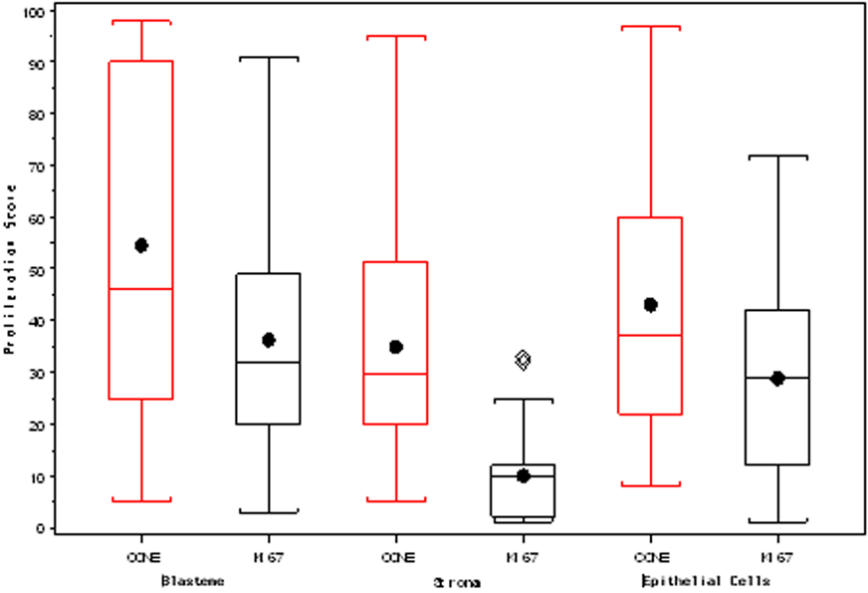
Box-Plot of the median Ki-67 and cyclin E *SI* in the different components of postchemotherapy nephroblastoma.

We determined Ki-67 and cyclin E staining index (*SI*) in all histological components when present (Table 1). The median *SI* for Ki-67 and cyclin E were 33% and 46% respectively in blastemal cells, 10% and 30% in stromal cells, 29.5% and 37% in epithelial cells (Figure 1).

**Table 1:** Ki-67 and cyclin E staining index (*SI*) in 54 postchemotherapy nephroblastoma. For each patient, age at surgery, sex, clinical features, tumor local stage and subtype, metastases and follow-up were specified. Median *SI* are in bold.

Tumor	Age (months)	Sex	Clinical features	Local stage	Type	Metastases	Outcome	Ki-67 *SI*			Cyclin E *SI*		
								blast	strom	epith	blast	strom	epith
T308B	42	M	Bilateral WT	II	diffuse anaplasia	no	alive	78	25	62	98	95	89
T187	48	F		II	diffuse anaplasia	yes (stage IV)	alive	42	12	26	96	37	80
T281	49	F		II	diffuse anaplasia	yes (stage IV)	alive	70	24			30	
T220	78	F		III	diffuse anaplasia	yes (stage IV+relapse)	dead	55		57	75		97
T284	50	F		III	diffuse anaplasia	yes (stage IV+relapse)	dead	71		29	93		63
								**70**	**24**	**43**	**94.5**	**37**	**84.5**
T207B	36	M	Bilateral WT	I	blastemal	no	alive	15			20		
T211	10	F		I	blastemal	no	alive	27			40		
T248	69	F		II	blastemal	no	alive	36			90		
T254	40	M		II	blastemal	no	alive	20			32		
T257	29	F	Hemihypertrophy	I	blastemal	no	alive	24			13		
T263	163	F		II	blastemal	no	alive	11			70		
T270B	12	F		III	blastemal	no	alive	36			5		
T279	45	F		II	blastemal	no	alive	40			90		
								**25.5**			**36**		
T96	16	M	Beckwith Wiedemann	I	mixed	no	alive	12		3	24		10
T100	46	M		II	mixed	no	alive		10	72		48	60
T163	16	F	Proteinuria	II	mixed	no	alive		32	21		65	
T189	29	M		II	mixed	no	alive	25	12		30	20	
T196	26	F		II	mixed	no	alive	20			90		
T200	56	F	Bilateral WT	I	mixed	no	alive	46	33	36	25	26	25
T204	21	M	Denys-Drash/Bilateral WT	II	mixed	no	alive		17			20	
T213A	35	F	Perlman/Bilateral WT	III	mixed	no	alive	5	5	30	20	5	20
T231	38	F		II	mixed	no	alive	30	4		61	21	
T246	14	F		II	mixed	no	alive	41			90		
T255	13	M		I	mixed	no	alive	30	10	34	33	33	34
T261	35	M	Beckwith Wiedemann	II	mixed	no	alive		12	20		11	75
T268	14	F	Bilateral WT	I	mixed	no	alive	3	1	10	6	8	14
T271A	12	M	Hypospadias/Bilateral WT	II	mixed	no	alive	33	6	43	44	26	33
T275	21	F		I	mixed	no	alive	40		48	60		60
T308A	17	M	Bilateral WT	II	mixed	no	alive	23	10	15	33	5	30
T400	23	F		III	mixed	no	alive	17	2	46	25	23	22
T401	31	F		III	mixed	no	alive	45	11	34	35	30	30
T402	23	M		III	mixed	no	dead	69	20	41	80	61	72
T404	40	M		II	mixed	no	alive	23		11	20		8
T405	39	M	Hemihypertrophy	III	mixed	no	alive	14	2	1	84	36	40
T406	82	F	Hemihypertrophy	III	mixed	no	alive	40	2,3	13	87	24	36
T202	20	M	Bilateral WT	I	mixed	yes (relapse)	alive	60	10	50	60	55	60
T212	18	M		I	mixed	yes (relapse)	alive	61			95		
T310	17	M		III	mixed	yes (relapse)	alive	91	2	60	95	70	47
T218	38	M		III	mixed	yes (stage IV+relapse)	alive	60			82		
T245	43	M		I	mixed	yes (stage IV+relapse)	alive	70			88		
								**33**	**10**	**34**	**60**	**26**	**33.5**
T201	10	M		II	stromal	no	alive		20	40		60	80
T209	8	F		II	stromal	no	alive		11			23	
T247	47	M		I	stromal	no	alive		2			12	
T269	10	F		I	stromal	no	alive		4			69	
T272	14	F		II	stromal	no	alive	8	2	8	25	32	20
T280	17	F	WAGR	I	stromal	no	alive	35	6	12	35	10	30
T403	35	F		I	stromal	no	alive	28	5	12	NI	NI	NI
T234	36	M		I	stromal	yes (stage IV)	alive		1,5			47	
T262	33	F	Bilateral WT	I	stromal	yes (relapse)	dead		5	11		29	51
								**28**	**5**	**12**	**30**	**30.5**	**40.5**
T219	11	F		I	epithelial	no	alive			32			38
T238	7	M		I	epithelial	no	alive			29			21
										**30,5**			**29,5**
T94	56	F		I	regressive	no	alive	5			13		
T264	70	F		III	regressive	no	alive	14			46		
T216	73	M		III	regressive	yes (stage IV)	alive		12			84	
								**9,5**			**29,5**		

blast = blastemal cells, strom = stromal cells, epith = epithelial cells, NI = noninterpretable

**Figure 2:**
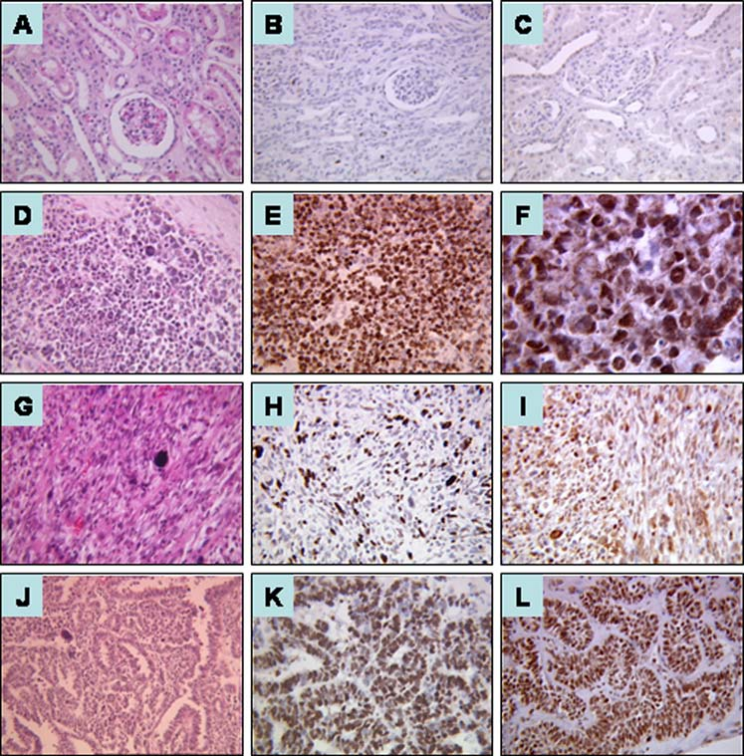
Hematoxylin and eosin staining (*H&E*), Ki-67 and cyclin E immunostaining in normal kidney and diffuse anaplastic type nephroblastoma. Normal kidney (A, *H&E*) shows only scattered positive nuclei in tubules after using Ki-67 antibody (B) and no positive nuclei after using cyclin E antibody (C). Original magnification, ×40. Diffuse anaplastic nephroblastoma with anaplastic cells in the blastemal (D), stromal (G) and epithelial (J) component (*H&E*; original magnification, ×40). Immunostaining shows numerous blastemal positive cells (E,F), stromal (H,I) and epithelial cells (K,L) using Ki-67 (E,H,K) and cyclin E (F,I,L). Original magnification, ×40 for E, H, I, K, L, ×100 for F.

In normal kidney parenchyma adjacent to the tumor, only scattered Ki-67 positive nuclei were present in the renal tubules, whereas we did not observe positive cyclin E nuclear staining (Figure 2A–C).

**Figure 3:**
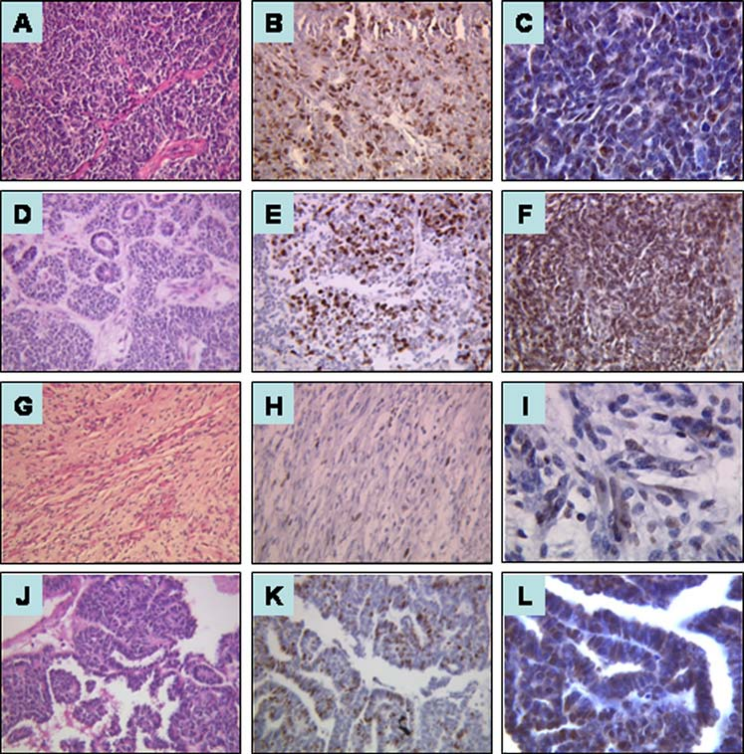
Hematoxylin and eosin staining, Ki-67 and Cyclin E immunostaining in nonanaplastic nephroblastoma. Examples of a blastemal type nephroblastoma (A, *H&E*) with a median *SI* of 25,5% for Ki-67 (B) and of 36% for cyclin E (C), of a blastemal component of a mixed type nephroblastoma (D, *H&E*) with a median *SI* of 33% for Ki-67 (E) and of 60% for cyclin E (F), of a stromal type nephroblastoma (G, *H&E*), with a median *SI* of 5% for Ki-67 (H) and of 30,5% for cyclin E (I), of an epithelial type nephroblastoma (J, *H&E*), with a *SI* of 30% for Ki-67 (K) and for cyclin E (L). Original magnification, ×40 for all panels except C, I, L (×100).

Ki-67 and cyclin E *SI* were then analyzed according to the histological type. Median *SI* are shown in Table 1 and are illustrated in Figures 2 and 3:

In anaplastic nephroblastoma, the median *SI* in the blastemal component (Fig. 2D) was 70% for Ki-67 (range, 42%–78%) (Fig. 2E) and 94.5% for cyclin E (range, 75%–98%) (Fig. 2F). In the stromal cells (Fig. 2G), it was 24% for Ki-67 (range, 12%–25%) (Fig. 2H) and 37% for cyclin E (range, 30%–95%) (Fig. 2I). In the epithelial cells (Fig. 2J), it was 43% for Ki-67 (range, 26%–62%) (Fig. 2K) and 84.5% for cyclin E (range, 63%–97%) (Fig. 2L).

In blastemal type nephroblastoma (Fig. 3A), the median Ki-67 and cyclin E blastemal *SI* were 25.5% (range, 11%–40%) and 36% (range, 5%–90%) respectively (Fig. 3B–C). To see if chemotherapy influences Ki-67 or cyclin E expression, we analyzed five primary nephrectomy cases: the median *SI* was 49% for Ki-67 (range, 31%–80%) and 30% for cyclin E (range, 22%–92%) (data not shown).

In mixed type nephroblastoma (Fig. 3D), Ki-67 and cyclin E *SI* were assessed in the blastemal component in 23 cases and in the epithelial and stromal components in 19 cases. The median Ki-67 and cyclin E *SI* (Fig. 3E) were respectively 33% (range, 3%–91%) and 60% (range, 6%–95%) (Fig. 3F) in the blastemal component, 10% (range, 1%–33%) and 26% (range, 5%–70%) in the stromal component, 34% (range, 1%–72%) and 33.5% (range, 8%–75%) in the epithelial component.

In stromal type nephroblastoma (Fig. 3G), the median *SI* was 5% for Ki-67 (range, 1.5%–20%) (Fig. 3H) and 30.5% for cyclin E (range, 10%–69%) (Fig. 3I) in the stromal cells, 28% for Ki-67 (range, 8%–35%) and 30% for cyclin E (range, 25%–35%) in the blastemal component, 12% for Ki-67 (range, 8%–40%) and 40.5% for cyclin E (range, 20%–80%) in the epithelial cells.

In the two epithelial type nephroblastoma (Fig. 3J), Ki-67 *SI* were 29% and 32% (Fig. 3K). Cyclin E *SI* were 21% and 38% (Fig. 3L).

We observed no significant difference for Ki-67 and cyclin E blastemal *SI* in blastemal type nephroblastoma versus mixed type (*p* = 0.24 for Ki-67 and *p* = 0.43 for cyclin E). We also did not observe significant difference for Ki-67 and cyclin E stromal *SI* between mixed and stromal type nephroblastoma (*p* = 0.23 for Ki-67 and *p* = 0.61 for cyclin E).

### Correlation between Ki-67 and cyclin E expression

A correlation between Ki-67 and cyclin E *SI* was observed in blastemal (*ρ* = 0.68, *p*<10^−4^) and epithelial (*ρ* = 0.51, *p* = 0.01) components. In contrast, no correlation was observed for the stromal component (*ρ* = 0.15, *p* = 0.44).

### Correlation between Ki-67 and cyclin E expression and metastases

Univariate analysis showed prognostic significance of blastemal Ki-67 *SI* for metastases (*p*<10^−4^). Epithelial and stromal Ki-67 *SI* did not have any prognostic value (*p* = 0.29 and *p* = 0.90 respectively). In contrast, cyclin E *SI* showed prognostic significance for metastases in blastemal, stromal and epithelial cells (*p* = 0.002, *p* = 0.03 and *p* = 0.01 respectively). Multivariate analysis could not be performed because of the low frequency of events. Table 2 summarizes the main data in patients with and without metastasis.

**Table 2:** Clinical, histological and immunohistochemical characteristics of patients with nephroblastoma with or without metastasis.

*Variables*	Patients with metastases (N = 12)	Patients without metastasis (N = 42)
***Age*** (months)	40.5* (17–78)	26* (7–163)
***Male***	7 (58%)	17 (40%)
***Preoperative chemotherapy***	12 (100%)	42 (100%)
***Sub-type NB:***		
Anaplastic	4 (33%)	1 (2%)
Blastemal	0 (0%)	8 (19%)
Epithelial	0 (0%)	2 (5%)
Mixed	5 (42%)	22 (52%)
Regressive	1 (8%)	2 (5%)
Stromal	2 (17%)	7 (17%)
***Pathological local staging:***		
I	5 (42%)	15 (36%)
II	2 (17%)	19 (45%)
III	5 (42%)	8 (19%)
***Death***	3 (25%)	1 (2%)
***Blastemal cells*** * (SI: median, range)*		
Ki-67	61 (42–91)	26 (3–78)
Cyclin E	90.5 (60–96)	35 (5–98)
***Stromal cells*** * (SI: median, range)*		
Ki-67	10 (1.5–24)	10 (1–33)
Cyclin E	47 (29–84)	25 (5–95)
***Epithelial cells*** *(SI: median, range)*		
Ki-67	39.5 (11–60)	29.5 (1–72)
Cyclin E	61.5 (47–97)	31.5 (8–89)

* significant difference between median age of patients with and without metastasis (*p* = 0.04)

**Figure 4:**
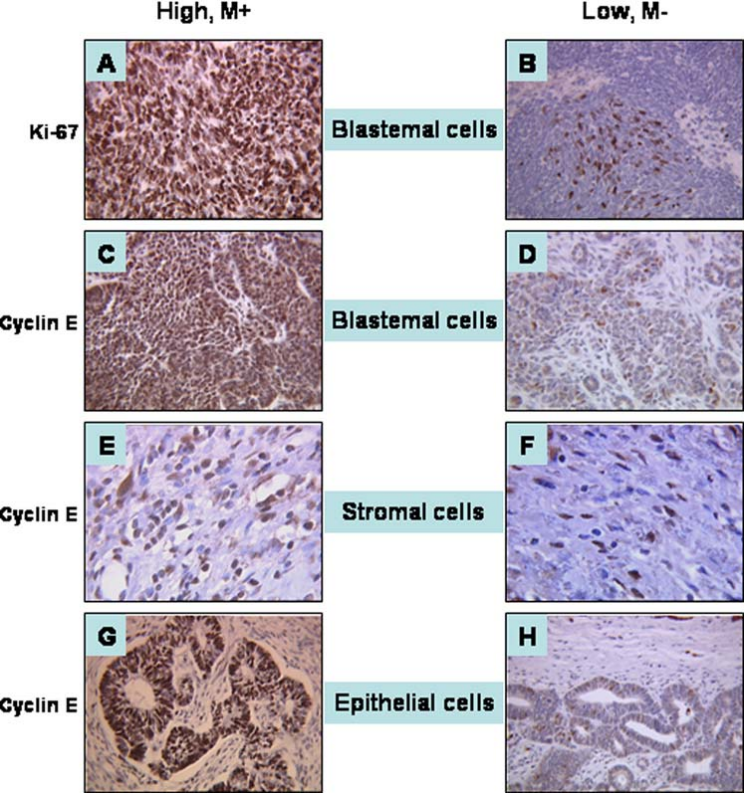
Immunostaining in mixed type nephroblastoma with or without associated metastasis. Example of high Ki-67 (A) and high cyclin E *SI* (C,E,G) in the blastemal (A,C), in the stromal (E) or epithelial (G) component of a patient with a stage I mixed type nephroblastoma and pulmonary metastases (M+) in comparison with a patient with a stage III mixed type nephroblastoma without metastasis (M-) and low Ki-67 (B) and cyclin E (D,F,H) *SI* in corresponding components.

When we focused on intermediate-risk nephroblastoma, the median blastemal Ki-67 *SI* was 61% (range, 60%–91%) for cases with metastases (n = 8) whereas it was 25% (range, 3%–69%) for cases without metastasis (n = 33). The median blastemal, stromal and epithelial cyclin E *SI* were also higher in nephroblastoma with metastases: 88% (range, 60%–95%), 55% (range, 29%–84%) and 51% (range, 47%–60%) respectively versus 34% (range, 6%–90%), 24% (range, 5%–69%) and 30% (range, 8%–80%) in nephroblastoma without metastasis. Figure 4 shows a representative pattern of Ki-67 and cyclin E *SI* in intermediate-risk mixed type tumors with (Fig. 4A,C,E and G) or without metastasis (Fig. 4B,D,F and H).

Using the Classification and Regression Trees (CART) procedure, we developed cutoff scores to predict metastases focusing on intermediate-risk nephroblastoma. We found a cutoff of 40% for blastemal Ki-67 (sensitivity, 0.75; specificity, 0.85), 50% for blastemal cyclin E (sensitivity, 0.67; specificity, 0.79), 28% for stromal cyclin E (sensitivity, 0.58; specificity, 0.79) and 44% for epithelial cyclin E, (sensitivity, 0.5; specificity, 0.87).

## Discussion

Our study showed that a high cyclin E *SI* in all components of nephroblastoma was correlated with tumor aggressiveness and metastases. The three histological components of nephroblastoma (blastemal, epithelial and stromal) have different proliferating potential. Furthermore, each component may have different proliferating activity in each different histological type of nephroblastoma. For example, blastemal cells may belong to the blastemal component of high-risk blastemal predominant nephroblastoma or to the blastemal component of an intermediate-risk histological type. However, in this latter type, such blastemal component can be aggressive too. This hypothesis is supported by the fact that distant metastases can develop in cases of stage I localized nephroblastoma, with intermediate-risk histology. Thus, markers enabling identification of aggressive component are of great interest.

Numerous studies have been reported which analyze the expression of genes at the RNA or protein level in order to detect correlations with adverse outcome [3], [4], [13], [14], [15], [16], [17]. Potential markers included proliferation markers, apoptosis-associated molecules, growth factors and cell adhesion molecules. For some of these molecules, i.e. the antiapoptotic factor Bcl-2 [18], TGFα [19], the angiogenic growth factor VEGF and its receptor Flt-1 [20], IGFIR (type I insulin-like growth factor receptor) [14], blastemal staining was indicative of clinical progression but epithelial staining has no prognostic value. To date, among the predictive markers identified, no immunohistochemistry staining has proven to be relevant in the three histological components of nephroblastoma.

It is well established that characterization of clinical relevant prognostic markers will require studies of combination of markers. However, difficulties in validating molecules previously reported [4] highlight the fact that the first step that consists of identifying single reliable markers, which could be further included in large association studies, must go on.

In this study, we analyzed two cell-cycle markers, Ki-67 and cyclin E, using immunohistochemistry in different components of postchemotherapy nephroblastoma histologically classified according to the SIOP classification. We determined Ki-67 *SI* in the three components of nephroblastoma and found the highest values in blastemal and epithelial components, as previously reported [21]. The clinical value of proliferation markers such as Ki-67 in nephroblastoma is still subject to debate. Nagoshi et al. [22] did not find any relationship between proliferation rates and patient survival whereas other studies [21], [23] showed that blastemal Ki-67 is a prognostic factor in pretreated nephroblastoma. Our study demonstrates that Ki-67 *SI* in blastemal cells is a highly significant indicator of metastases whereas it has no prognostic value in epithelial nor in stromal cells. When focusing only on intermediate-risk nephroblastoma, we still found higher values of blastemal Ki-67 *SI* in nephroblastoma with metastases in comparison with those without metastasis.

Cyclin E, a G1-cyclin, is a marker of cell-cycle progression. Overexpression of cyclin E has been demonstrated to be an indicator of poor prognosis in many cancers [9], [10], [11], [12]. In the cyclin E promoter, several WT1 binding sites have been identified and WT1, which is the tumor suppressor gene defective in some nephroblastoma, has a repressive effect on cyclin E expression [24]. But, to our knowledge, cyclin E expression has never been studied in nephroblastoma. Faussillon et al. reported overexpression of another cyclin, cyclin D2 (CCND2), in 86% of Wilms' tumor, supporting the idea that alteration at the G1/S cell cycle control point is of biological significance in nephroblastoma [25]. However they found no association between relapse and cyclin D2 overexpression.

We used an antibody that can recognize both full-length and lower molecular weight isoforms of cyclin E. The latter are of particular interest because recent data demonstrate that they are specific to tumor cells [26], [27]. We found the highest values for cyclin E *SI* in blastemal cells with prognostic significance for distant metastases. Cyclin E is also largely expressed in epithelial and stromal cells where it has a prognostic value too. Our study demonstrates that increased cyclin E expression is associated with distant metastases for whichever histological component of nephroblastoma it is assessed in. Moreover when focusing on intermediate-risk nephroblastoma, the ones with metastases have higher cyclin E *SI* in the three components than those without metastasis.

We observed a correlation between Ki-67 and cyclin E levels in blastemal and epithelial components. These data suggest that high cyclin E *SI*, at least in these two components, may reflect increased proliferative activity. However, we found a decreased blastemal proliferation, as assessed by Ki-67 after chemotherapy, in accordance with another study [21] whereas no variation of blastemal cyclin E *SI* was found, suggesting that cyclin E is not only a proliferation marker. Some studies assessing Ki-67 and cyclin E activities in breast cancer also concluded that cyclin E provides additional information in that it represents a marker for both proliferation and oncogenesis [28], [29]. Porter et al. [28] reported a subset of tumors with discordant cyclin E expression and proliferation index (i.e., high cyclin E and low Ki-67 levels) showing a strong association with mortality. Moreover, cyclin E staining detects tumor cells committed to cell division (late G1 and beyond). Such improvement in tumor phenotyping may enable prediction of responsiveness to chemotherapy targeted at cells in S and M phases [29]. Even if the basis for cyclin E prognostic significance is not known, a major hypothesis is that cyclin E overexpression reflects alterations in any part of the p16–cyclinD–Rb–E2F pathway, which is mutated in most cancers. Thus, cyclin E expression may be a single convenient marker of alterations occurring in a very complex pathway [8].

Using a CART procedure, we determined cyclin E and Ki-67 cutoff scores for metastasis prediction. A high specificity was found for Ki-67 and cyclin E, but sensitivity was higher for blastemal Ki-67 than for cyclin E. Optimal determination of these cutoffs needs a larger cohort of patients and a centralized immunohistochemical study to avoid technical variations.

As a summary, blastemal, epithelial and stromal cyclin E *SI* and blastemal Ki-67 *SI* are significant determinants of distant metastases. Accurate postoperative chemotherapy is based on histology and staging. Risk histological stratification could also include immunohistochemical analyses to better explore aggressiveness in the different components of nephroblastoma. Cooperative larger studies focusing on intermediate-risk nephroblastoma are needed to determine if these two markers should be considered for stratification in future trials.

## Materials and Methods

### Patients

This retrospective study included 54 tumors removed from 53 patients (23 males and 30 females). Eight of these patients were syndromic: one WAGR syndrome, one Denys–Drash syndrome, one Perlman syndrome and five Beckwith–Wiedemann syndrome or hemihypertrophy patients. The patients were seven to 163 months of age; the median age at surgery was 32 months.

All patients were treated by neoadjuvant chemotherapy before nephrectomy. All the slides were reviewed by the French National referent for SIOP-2001 Nephroblastoma Study (LBG) and local pathologists. Exclusion criteria were: low-risk tumors and regressive type nephroblastoma with more than 90% of chemotherapy-induced changes.

Thirteen high-risk nephroblastoma (5 anaplastic and 8 postchemotherapy blastemal types) and 41 intermediate-risk nephroblastoma (9 stromal, 3 regressive, 27 mixed and 2 epithelial types) were studied. Pathological local staging was: stage I (n = 20), stage II (n = 21), stage III (n = 13). Three blastemal type and 2 mixed type from primary nephrectomy cases (4 under seven months of age and one with a highly cystic tumor) were also separately analyzed for comparison.

Forty-six tumors were removed from patients with a localized disease at diagnosis (i.e. unilateral or bilateral nephroblastoma, without metastasis), and 8 tumors from patients with metastases at diagnosis (clinical stage IV). Among the 46 patients with localized disease, 4 developed metastases after nephrectomy. The median follow-up was 57 months.

### Specimen characteristics

This retrospective study was performed on samples obtained from the SIOP-2001 nephroblastoma protocol and conducted according to the French legislation with consent from the donors obtained for collection of this material. All nephrectomy specimens were fixed in 10% buffered formalin. For each tumor, at least two paraffin-embedded tissue sections, representative of the global histology, were selected for immunohistochemical analysis.

### Antibodies

Primary antibodies were: mouse monoclonal anti-human Ki-67 antigen (clone MIB-1, DakoCytomation, Glostrup, Denmark) and rabbit polyclonal affinity purified antibody against a peptide mapping at the C-terminus of the human cyclin E (C-19, Santa Cruz Biotechnology, Santa Cruz, CA).

### Immunohistochemical staining

After paraffin removal, sections were heated in a water bath in sodium citrate buffer 0.1 M, pH 6.0 for 40 min for antigen retrieval. Endogenous peroxidase activity was quenched with a 3% hydrogen peroxide solution. The slides were then incubated with primary antibody: anti-MIB-1 (1∶50) for 30 min at room temperature or anti-cyclin E antibody (1∶80) overnight at 4°C. For MIB-1 primary antibody detection, incubation with biotinylated sheep anti-mouse immunoglobulin for 30 min followed by incubation with horseradish peroxidase coupled streptavidin for 30 min was performed. For anti-cyclin E primary antibody detection, the DAKO EnVision system (DakoCytomation) was used (30 min). In both cases, 3,3′-diaminobenzidine was used as a substrate. Sections were counterstained with hematoxylin.

### Quantification of labeled cells

Immunostaining was assessed by two independent observers blinded to clinical outcome and local staging. Staining was evaluated in tumor cells and adjacent normal kidney. Only nuclear staining was considered as positive. The intensity of staining was not recorded. For anti-cyclin E antibody, the positive control was a breast adenocarcinoma [28]. We recorded at high magnification the number of cyclin E and Ki-67 positive nuclei in at least 500 cells in representative fields. The average percentage of stained tumor cells was calculated and assigned to a staining index (*SI*).

### Statistical analysis

Results are expressed as median and extreme values for quantitative variables, and numbers (percentages) for qualitative variables. Relationships between metastasis and age, Ki-67 *SI*, cyclin E *SI* were studied by non-parametric Wilcoxon Rank Sum test. Association between Ki-67 and cyclin E *SI* was studied by Spearman correlation statistics.

We developed cutoff scores to predict metastasis with Ki-67 and cyclin E in the different components of nephroblastoma by the CART (Classification and Regression Trees) procedure [30]. All tests were two tailed. Statistical analyses were performed using the SAS 9.1 (SAS Inc, Cary, NC) software package, with *p*<0.05 considered as statistically significant.
